# Molecular Network Analysis of Circulating microRNAs Highlights miR-17-5p and miR-29a-3p as Potential Biomarkers of Aortic Valve Calcification

**DOI:** 10.3390/ijms262210813

**Published:** 2025-11-07

**Authors:** Antonella Galeone, Arianna Minoia, Michele Braggio, Mattia Cominacini, Maria Grazia Romanelli, Luca Dalle Carbonare, Giuseppe Faggian, Giovanni Battista Luciani, Maria Teresa Valenti

**Affiliations:** 1 Department of Surgery, Dentistry, Pediatrics and Gynecology, Division of Cardiac Surgery, University of Verona, 37126 Verona, Italy; 2 Department of Engineering for the Innovation Medicine, University of Verona, 37100 Verona, Italy; 3 Department of Neurosciences, Biomedicine and Movement Sciences, University of Verona, 37100 Verona, Italy

**Keywords:** calcific aortic valve disease, micro-RNA, biomarker

## Abstract

Calcific aortic valve disease (CAVD) is characterized by progressive valve remodeling and calcification. Moreover, microRNAs (miRNAs) are emerging as key regulators of cardiovascular pathology and potential circulating biomarkers. We performed high-throughput miRNA profiling in calcified aortic valve tissue and matched patient serum samples using an array that included 98 human miRNAs. Expression data were log10-transformed and filtered to identify biologically relevant miRNAs. Shared miRNAs between tissue and serum were further validated by quantitative real-time polymerase chain reaction (qRT-PCR) in patients and healthy controls. Of the 49 actively expressed miRNAs, 18 were shared between valve tissue and serum. Thus, qRT-PCR validation revealed significant downregulation of miR-17-5p and miR-29a-3p in CAVD patient serum compared to controls. These results indicate that disease-associated miRNA alterations in calcified valves are mirrored in circulation. miR-17-5p and miR-29a-3p represent promising circulating biomarkers for CAVD, reflecting underlying pathological remodeling and extracellular matrix dysregulation. Our findings provide a framework for non-invasive monitoring of valve calcification and highlight miRNA-mediated pathways as potential therapeutic targets.

## 1. Introduction

Calcific aortic valve disease (CAVD) represents the most common valvular heart disorder in industrialized countries, characterized by progressive fibro-calcific remodeling of the aortic valve leaflets leading to stenosis and impaired cardiac function [1]. Once considered a passive, degenerative process associated with aging, valve calcification is now recognized as an active and highly regulated biological process, involving osteogenic trans-differentiation of valvular interstitial cells, chronic inflammation, lipid infiltration, extracellular matrix remodeling, and apoptosis [1,2,3,4,5]. Despite its high prevalence and clinical impact, effective pharmacological strategies to prevent or slow disease progression are currently lacking, making the identification of novel biomarkers and molecular targets a crucial research priority. In recent years, microRNAs (miRNAs), small non-coding RNAs that regulate gene expression post-transcriptionally, have emerged as key players in cardiovascular pathophysiology [6]. Specific miRNAs have been implicated in endothelial dysfunction, inflammation, and osteogenic differentiation, all processes that contribute to valvular calcification [7,8]. Importantly, miRNAs can be detected not only in diseased valve tissue but also in circulating blood, suggesting their potential as minimally invasive biomarkers for disease diagnosis, monitoring, and risk stratification [9]. Several studies have reported dysregulated expression of distinct miRNAs in patients with valvular heart disease, highlighting their dual role as mediators and indicators of pathological remodeling [10]. However, the relationship between miRNA expression in valvular tissue and their circulating levels in patients with calcific valve disease remains poorly understood. Therefore, a better understanding of this relationship may help elucidate the molecular mechanisms driving disease progression and identify clinically relevant biomarker signatures. The present study aims to compare miRNA expression profiles in calcified valve tissue and serum samples from patients with CAVD. By correlating tissue and circulating miRNA patterns, we should provide new insights into the molecular pathways underlying CAVD and explore the feasibility of circulating miRNAs as potential biomarkers reflecting local pathological processes in the valve.

## 2. Results

### 2.1. MiRNA Expression Profiling in Calcified Aortic Valves and Patients Sera

**Figure 1:**
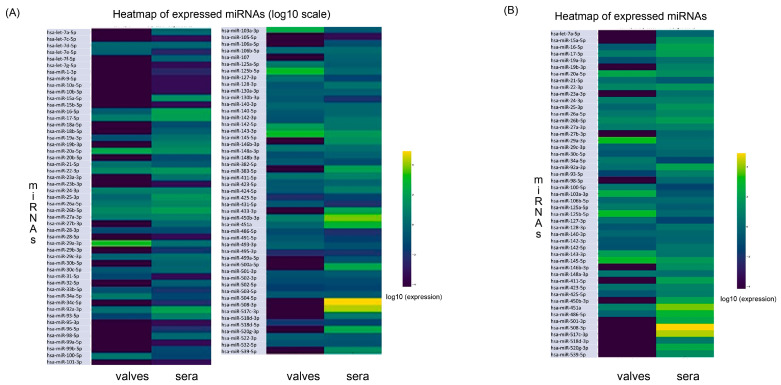
Schemes follow the same formatting. (**A**) Heatmap showing the expression levels of all 98 miRNAs identified in calcified aortic valve tissue and corresponding serum samples from patients. Expression values were log10-transformed to normalize for differences in signal intensity across samples. Each row represents a single miRNA, and each column corresponds to an individual sample (valve or serum). (**B**) Heatmap showing the expression profiles of the 49 miRNAs retained after applying an expression threshold (log10-transformed values > 1 in at least one condition) to the initial dataset of 98 miRNAs. The color scale represents expression values transformed using base-10 logarithms (lighter colors = higher values, darker colors = lower values).

To obtain a comprehensive overview of miRNA expression associated with valvular calcification, RNA was extracted from both calcified aortic valve tissue and corresponding serum samples from patients undergoing aortic valve replacement surgery. Thus, a high-throughput miRNA array including 98 unique human miRNAs was performed. Given the broad dynamic range of expression values observed across the two sample types, raw data were log10-transformed to facilitate inter-sample comparison and visualization. The heatmap including all 98 miRNAs revealed distinct expression clusters separating valve tissue from serum, suggesting the presence of tissue-enriched as well as systemically detectable miRNA populations. In particular, several miRNAs showed high expression levels specifically in valve tissue, whereas others displayed a more homogeneous distribution across both tissue and serum samples (Figure 1A). Therefore, to focus the analysis on biologically meaningful miRNAs, we applied a filtering threshold based on expression levels >1 in at least one of the two experimental conditions (valve or serum). This selection criterion reduced the dataset from 98 to 49 expressed miRNAs, which were represented in a dedicated heatmap (Figure 1A,B). This intermediate filtering step allowed for a more precise identification of miRNAs actively expressed in the pathological context of calcific aortic valve disease, excluding l and 1 Bow-abundance signals and background noise.

### 2.2. Shared miRNAs Between Valve Tissue and Serum

Among the 49 expressed miRNAs, 18 miRNAs were shared between calcified valve tissue and serum samples, indicating a molecular overlap between local valvular processes and systemic circulation. These shared miRNAs were: miR-17, miR-20a, miR-22, miR-24, miR-25, miR-26a, miR-26b, miR-27a, miR-29a, miR-92a, miR-106b, miR-125b, miR-140, miR-143, miR-145, miR-148a, miR-423, and miR-451a (Supplemental Table S1). The presence of these miRNAs in both samples supports the hypothesis that disease-associated miRNA alterations in the valve are reflected at the systemic level, potentially enabling their use as circulating biomarkers for disease detection and monitoring.

### 2.3. Pathway Enrichment Analysis of Shared Differentially Expressed miRNAs

**Figure 2:**
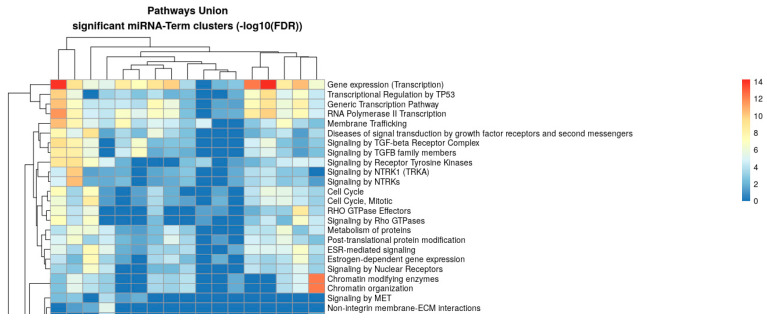
Heatmap showing the union of significantly enriched pathways based on experimentally validated miRNA–target interactions analyzed with the DIANA-miRPath v4.0 platform (TarBase v8 and Reactome Pathway Union database, miRBase release v22.1, accessed on 20 August 2025, at http://62.217.122.229:3838/app/miRPathv4). Pathway enrichment significance was calculated using FDR-corrected *p*-values (threshold 0.05) and expressed as −log10(FDR). Hierarchical clustering was performed on significant miRNA–term associations to identify shared biological processes. Red indicates the highest level of significance, while blue represents lower significance levels.

To explore the biological functions potentially regulated by the identified miRNAs, we performed pathway enrichment analysis using the DIANA-miRPath v4.0 platform (TarBase v8 and Reactome Pathway Union database, miRBase release v22.1, accessed on 20 August 2025, at http://62.217.122.229:3838/app/miRPathv4). Experimentally validated miRNA–target interactions were retrieved and mapped to Reactome pathways (miRBase release v22.1). Enrichment significance was assessed using FDR-corrected *p*-values with a threshold of 0.05. The analysis revealed significant enrichment of multiple pathways, including gene expression (transcription), transcriptional regulation by TP53, TGF-β signaling, receptor tyrosine kinase signaling, cell cycle regulation, Rho GTPase effectors, chromatin organization, and membrane trafficking (Figure 2). The heatmap shows the union of enriched pathways across significant miRNA-term clusters, with enrichment scores represented as −log10(FDR).

Moreover, KEGG pathway enrichment analysis using the pathways union method in DIANA-miRPath v4.0 revealed several biological processes involving differentially expressed miRNAs (Table 1).

**Table 1:** Kyoto encyclopedia of genes and genomes and gene ontology pathways regulated by the differentially expressed microRNAs. The list of target genes of the analyzed miRNAs is provided in Supplementary Table S2.

Term Name	Term Genes	miRNAs (n)	miRNA Names	Merged *p*-Value	Merged FDR
Signaling by TGFB family members	104	11	hsa-miR-17-5p hsa-miR-20a-5p hsa-miR-22-3p hsa-miR-24-3p hsa-miR-25-3p hsa-miR-26a-5p hsa-miR-26b-5p hsa-miR-27a-3p hsa-miR-92a-3p hsa-miR-125b-5p hsa-miR-126b-5p	7.5 × 10^−61^	4.89 × 10^−59^
Signaling by WNT	366	7	hsa-miR-17-5p hsa-miR-20a-5p hsa-miR-22-3p hsa-miR-24-3p hsa-miR-29a-3p hsa-miR-92a-3p hsa-miR-125b-5p	1.68 × 10^−20^	1.68 × 10^−18^
PI3K-Akt signaling pathway	372	10	hsa-miR-17-5p hsa-miR-20a-5p hsa-miR-22-3p hsa-miR-24-3p hsa-miR-25-3p hsa-miR-26a-5p hsa-miR-26b-5p hsa-miR-27a-3p hsa-miR-29a-3p hsa-miR-145-5p	1.59 × 10^−29^	1.06 × 10^−28^
Signaling pathway regulating pluripotency of Stem Cells	156	10	hsa-miR-17-5p hsa-miR-22-3p hsa-miR-24-3p hsa-miR-26a-5p hsa-miR-26b-5p hsa-miR-27a-3p hsa-miR-29a-3p hsa-miR-92a-3p hsa-miR-125b-5p hsa-miR-106b-5p	7.16 × 10^−27^	4.08 × 10^−26^

To further characterize the biological processes regulated by the identified miRNAs, enrichment analysis was also performed using the Gene Ontology (GO) terms Pathway Union database through miRPath v4 (TarBase v8). Among the significantly enriched GO terms, heart development showed a strong association (FDR = 2.40 × 10^−29^; *p*-value = 2.53 × 10^−30^), involving 7 miRNAs: *hsa-miR-17-5p*, *hsa-miR-22-3p*, *hsa-miR-24-3p*, *hsa-miR-27a-3p*, *hsa-miR-29a-3p*, *hsa-miR-125b-5p*, and *hsa-miR-106b-5p* (Table 2).

### 2.4. Downregulation of miR-17-5p and miT-29a-3p in Patient Serum as Potential Circulating Biomarkers for Detection of Valve Calcification

**Figure 3:**
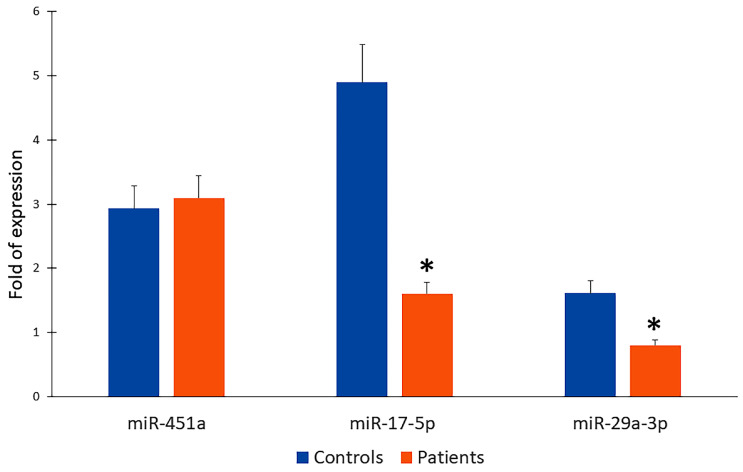
MiR-451a, miR-17-5p, and miR-29a-3p qRT-PCR in controls and patients; * *p* < 0.005.

Based on the array profiling data and the pathway association miR-17-5p and miR-29a-3p were selected for independent validation by quantitative real-time polymerase chain reaction (qRT-PCR) in serum samples from patients with calcified aortic valves and healthy control subjects. We also analysed miR-451a which was express in array profiling but no KEGG pathways were enriched by genes targeted by miR-451a alone or in combination with other studied miRs. Thus, the expression levels of miR-451a were comparable between patients and healthy controls, showing mean fold changes of 3.20 and 2.91, respectively, and no statistically significant difference (*p* = 1) (Figure 3). These results suggest that miR-451a may not be directly involved in the pathological mechanisms of valve calcification or may act as a stable circulating miRNA.

In contrast, miR-17-5p expression was significantly downregulated in patient serum compared to controls (1.70 vs. 4.72-fold; *p* = 0.014) (Figure 3). Similarly, miR-29a-3p was also significantly reduced in patients compared with controls (0.86 vs. 1.79-fold; *p* = 0.014) (Figure 3). Overall, these findings suggest that miR-17-5p and miR-29a-3p could be considered potential circulating biomarkers for CAVD characterization.

## 3. Discussion

In this study, we performed a comprehensive analysis of miRNA expression in calcified aortic valve tissue and corresponding patient serum, aiming to identify molecular signatures reflective of valvular pathology and potential circulating biomarkers. Our high-throughput profiling revealed 98 miRNAs expressed across tissue and serum, with clear clustering distinguishing valve-enriched from systemically detectable miRNAs. Application of an expression threshold allowed refinement of the dataset to 49 actively expressed miRNAs, facilitating the identification of biologically relevant candidates while minimizing background noise. Among these, 18 miRNAs were consistently shared between valve tissue and serum, including miR-17, miR-20a, miR-22, miR-24, miR-25, miR-26a/b, miR-27a, miR-29a, miR-92a, miR-106b, miR-125b, miR-140, miR-143, miR-145, miR-148a, miR-423, and miR-451a. The presence of these miRNAs in both compartments suggests that alterations occurring within the calcified valve are reflected in the systemic circulation, supporting their potential as minimally invasive biomarkers for disease detection and monitoring. Pathway enrichment analysis revealed that several key signaling cascades are regulated by the differentially expressed miRNAs identified in this study. In particular, the most significantly enriched pathways included the TGF-β signaling pathway (FDR = 4.89 × 10^−59^), Wnt signaling pathway (FDR = 1.68 × 10^−18^), and PI3K-Akt signaling pathway (FDR = 1.06 × 10^−28^). These pathways are known to play fundamental roles in cardiovascular homeostasis, tissue remodeling, and pathological calcification processes [1,11,12,13,14]. Several miRNAs—including hsa-miR-17-5p, hsa-miR-20a-5p, hsa-miR-22-3p, hsa-miR-24-3p, hsa-miR-25-3p, hsa-miR-26a/b-5p, hsa-miR-27a-3p, hsa-miR-29a-3p, hsa-miR-92a-3p, and hsa-miR-125b-5p—were found to be commonly associated with these pathways, suggesting that they may act as central regulatory nodes in disease-relevant molecular networks. Of particular interest, the pathway regulating pluripotency of stem cells was also significantly enriched (FDR = 4.08 × 10^−26^), highlighting the potential involvement of miRNA-mediated mechanisms in modulating cellular differentiation and plasticity during disease progression. Although the pathway enrichment analysis strongly supports the involvement of the identified miRNAs in signaling pathways relevant to aortic valve calcification, experimental validation of their predicted target genes will be required to fully elucidate their mechanistic contribution. Due to the limited availability of biological material in the current study, this analysis could not be performed and will be prioritized in future investigations using additional patient samples. However, our findings are in line with previous reports indicating that reactivation of developmental signaling cascades contributes to the osteogenic trans-differentiation of valvular interstitial cells and extracellular matrix remodeling in calcific valve disease [15,16,17]. Complementary enrichment analysis using GO terms further strengthened these observations. “Heart development” emerged as the most significantly associated biological process (FDR = 2.40 × 10^−29^), involving seven miRNAs (hsa-miR-17-5p, hsa-miR-22-3p, hsa-miR-24-3p, hsa-miR-27a-3p, hsa-miR-29a-3p, hsa-miR-125b-5p, and hsa-miR-106b-5p). The strong association with this GO term suggests that the dysregulated miRNAs may participate in developmental programs that are aberrantly reactivated during the pathological calcification of aortic valves [18,19,20,21]. Based on their enrichment in key biological pathways, miR-17-5p and miR-29a-3p were selected for validation as potential circulating biomarkers. Specifically, both miRNAs presented strong enrichment in pathways relevant to disease pathogenesis (including pluripotency regulation in stem cells, WNT signalling, and heart development). Therefore, their validation was prioritized due to the combination of strong biological relevance and potential clinical utility. Quantitative real-time PCR confirmed a significant downregulation of both miR-17-5p (1.70-fold vs. 4.72-fold in controls, *p* = 0.004) and miR-29a-3p (0.86-fold vs. 1.79-fold in controls, *p* = 0.002) in patient serum. These findings support their potential role as non-invasive biomarkers for disease detection and characterization. Accordingly, it has been demonstrated that miR-17-5p can attenuate osteogenic differentiation and that vascular smooth muscle cells (VSMCs) transfected with a miR-17-5p mimic—thus enhancing its expression—exhibit a significant reduction in calcium deposition [22]. A decreased expression of miR-17-5p has also been reported in patients with bicuspid aortic valve (BAV), raising questions about its regulatory role in VSMC proliferation and valve development [23]. A reduction in miR-17a-5p could lead to enhanced calcification of the aortic valve by failing to suppress osteogenic pathways [24]. miR-17-5p is part of the miR-17-92 cluster which influences cell cycle progression and Notch signaling [25]. Reduced miR-17-5p could affect the expression of NOTCH1, a gene implicated in BAV pathogenesis [26]. By downregulating miR-17-5p, there may be increased expression of pro-calcific genes or dysregulated VSMC phenotypic switching, favoring a calcific environment or abnormal vascular remodeling [27]. In a chronic kidney disease (CKD) rat model, miR-17-5p has also been identified as a calcification-protective miRNAs that targets vascular endothelial growth factor A (VEGFA) signaling in CKD-driven vascular calcification [28]. Therefore, the reduced expression levels of miR-17-5p observed in CAVD may contribute to the development of calcification, suggesting a potential protective role for miR-17-5p in counteracting these processes. Regarding miR-29, it has been reported in a rat model that a miR-29a-3p mimic induces osteogenic differentiation, as demonstrated by the upregulation of RUNX2, COL1A1, and ALP compared to a negative control mimic. This suggests that miR-29a-3p enhances osteogenesis by activating key osteogenic genes [29]. In vitro studies showed that miR-29a-3p overexpression significantly attenuated VSMC calcification under high phosphate conditions, while VEGFA overexpression exacerbated calcification. Notably, miR-29a-3p counteracted calcification even in VEGFA-overexpressing cells, underscoring its protective role in vascular integrity [30]. However, miR-29 isoforms display pleiotropic and context-dependent functions, with their effects varying according to the cellular context (e.g., osteogenic versus inflammatory compartments), disease stage, and compensatory regulatory mechanisms. Notably, miR-29-3p has also been shown to suppress macrophage lineage commitment and exert anti-inflammatory effects [31]. Thus, in serum of CAVD patients, the observed downregulation of miR-29-3p may therefore reflect alterations in cellular composition, inflammatory signaling, or feedback regulation rather than a simple loss of a pro-osteogenic factor. Taken together, these findings highlight distinct and potentially complementary roles of miR-17-5p and miR-29-3p in the pathophysiology of valvular calcification, suggesting that their regulation is intricately linked to both osteogenic and inflammatory processes. In contrast, miR-451a, which was detected in the profiling data but not linked to any significantly enriched pathway, showed no significant difference between groups, suggesting a limited or indirect involvement in the disease process.

Overall, these results point to a coordinated dysregulation of miRNAs affecting multiple signaling pathways central to cardiovascular development and pathological remodeling. The identification of miR-17-5p and miR-29a-3p as significantly downregulated in patient serum further underscores their potential as biomarkers for CAVD [32,33,34,35].

## 4. Materials and Methods

This study was conducted in accordance with the Declaration of Helsinki and approved by the Ethics Committee of the Azienda Ospedaliera Universitaria Integrata of Verona (approval number: 13371; approval date: 13 March 2016). Informed consent was obtained from all participants in the study.

### 4.1. Aortic Valves and Sera

Human tricuspid aortic valves were obtained from four adult patients undergoing isolated surgical aortic valve replacement (SAVR) for severe calcific aortic stenosis. Inclusion criteria were: male sex, age < 75 years, tricuspid aortic valve, severe aortic stenosis requiring isolated SAVR, e-GFR ≥ 50 mL/min; exclusion criteria were: female sex, age > 75 years, BAV, concomitant coronary artery disease, mitral valve disease or aortic aneurysm, atrial fibrillation, e-GFR < 50 mL/min, diabetes, priori or ongoing neoplasia, prior or ongoing infective endocarditis, rheumatic disease, autoimmune disease, therapy with warfarin, DOACs, levothyroxine or steroids, enteral or parenteral nutrition. Aortic valve leaflets were collected during surgery immediately after valve leaflets removal, rinsed with saline solution (NaCl 0.9%) under sterile conditions and kept overnight in RNAlater^®^ (Qiagen, Hilden, Germany) at 4 °C and then frozen and stored at −80 °C until further use. Blood samples were collected from the same patients before surgery and from four age-matched healthy controls in serum separator tubes without anticoagulant. Samples were centrifuged at 1800× *g* for 15 min. The serum was aliquoted and stored at −80 °C until analysis.

### 4.2. Tissue Processing and Homogenization

Cardiac valve tissues were processed under sterile and RNase-free conditions. Before miRNA extraction, the tissues were homogenized using a mechanical disruption system- Precellys 24 (Bertin Technologies, Montigny-le-Bretonneux, France), to ensure complete tissue lysis. The homogenates were subsequently processed for downstream molecular analyses.

### 4.3. miRNA Extraction and Quantification

Circulating miRNAs were isolated from human serum samples using the miRNeasy^®^ Serum/Plasma Kit (Cat. Number 217204, Qiagen, Hilden, Germany) according to the manufacturer’s instructions. For cardiac valve tissues, miRNA was extracted using the miRNeasy^®^ Mini kit (Cat. Number 217004 Qiagen, Hilden, Germany) following the manufacturer’s protocol. miRNAs were quantified by NanoDrop™ spectrophotometer (LifeReal, Aurogene, Hangzhou Biotechnology, Hangzhou, China) and then 10 ng of the extracted miRNAs were reverse transcribed with a TaqMan™ Advanced miRNA cDNA Synthesis Kit (Cat. Number A28007, Thermo Fisher Corporation, Waltham, MA, USA) following the manufacturer’s instructions.

### 4.4. TaqMan Gene Expression Array

TaqMan Gene Expression Array analysis was performed using the TaqMan™ Advanced miRNA Human A 96-well Plates, standard-Panel 1,3,4 (Ref: A32026, Applied Biosystems, Thermo Fisher Scientific, Waltham, MA, USA) for both cDNA derived from serum and cardiac valve tissue. cDNA solution was prepared according to the manufacturer’s protocol. Then, the 96-well array Plate was centrifuged and sealed using the proper membrane (Opti-seal, AB analitica). The PCRs protocol was then performed on the LineGene 9620 Real-Time PCR System (Aurogene, Hangzhou Bioer Technology, Hangzhou, China) using the parameters listed in Table 3 below.

### 4.5. Gene Expression Analysis/ Real Time-PCR

The Real-Time PCR was performed using the TaqMan Fast Advanced Master Mix (Cat. Number 4444557, Thermo Fisher Scientific, Waltham, MA, USA) with the following commercially pre-designed probes-TaqMan Advanced miRNA assay (Cat. Number A25576, Thermo Fisher Scientific, Waltham, MA, USA) (Table 4).

The MicroAmp Optical 96-Well Reaction Plate (Applied Biosystems, Thermo Fisher Scientific, Waltham, MA, USA) was used to load 15 µL of PCR mix into the designed wells. Next, 5 µL (10 ng) of cDNA was added. Three copies of each sample were loaded. The plate was covered and sealed using the proper membrane (Opti-seal, AB analitica, Padova, Italy) following a brief centrifugation. The thermal protocol was performed on the LineGene 9620 Real-Time PCR System (Aurogene, Hangzhou Bioer Technology, China) using the parameters listed in Table 3. Each analysis was performed in triplicate and with Ct values averaged. miR-191-5p was used as housekeeping gene based on its stable expression across all samples. In addition, the choice of miR-191-5p is supported by published studies in mesenchymal stromal cells and human serum samples [36,37].

### 4.6. Pathway Enrichment Analysis

To explore the biological significance of the shared set of 18 differentially expressed miRNAs identified in calcified aortic valves and patient sera, pathway enrichment and functional annotation analyses were performed using publicly available bioinformatics tools. Experimentally validated miRNA–target interactions were analyzed using miRPath v4 DIANA-miRPath v4.0 [38], which integrates TarBase v8 for validated miRNA–gene interactions. The list of miRNAs identified in this study was submitted to miRPath v4 for pathway enrichment analysis using the Reactome Pathway Union database. miRBase release v22.1 was used as the reference for miRNA annotation. Enrichment significance was assessed using a false discovery rate (FDR)-corrected p-value threshold of 0.05. Results were expressed as −log10(FDR) values. Hierarchical clustering of significant miRNA–term associations was performed to identify shared biological processes among the input miRNAs. A heatmap was generated to visualize the most significantly enriched pathways miRNA-specific.

### 4.7. Statistical Analysis

Expression levels of miRNAs in healthy controls and patients were compared using the Mann–Whitney U test for independent samples, as the groups were independent and sample sizes were small (*n* = 4 per group). Data are presented as mean ± standard deviation (SD). A *p*-value of less than 0.05 was considered statistically significant. Statistical evaluations were conducted using GraphPad Prism (version 9).

## 5. Conclusions

In this study we demonstrate a clear overlap between miRNA signatures in calcified valve tissue and patient serum, with miR-17-5p and miR-29a-3p emerging as promising circulating biomarkers. These findings enhance our understanding of the molecular underpinnings of CAVD, pave the way for the development of non-invasive diagnostic and prognostic tools based on miRNA profiling, and highlight miRNA-mediated pathways as potential therapeutic targets.

Our study has some limitations. The sample size, while sufficient to identify robust changes in miRNA expression, warrants expansion in future studies to validate the utility of these miRNAs as predictive biomarkers across diverse patient cohorts. Additionally, functional studies are needed to delineate the mechanistic roles of miR-17-5p and miR-29a-3p in valve calcification and systemic cardiovascular remodeling.

## Figures and Tables

**Table 2:** GO terms enrichment analysis of identified miRNAs.

GO Term	No. of Genes	No. of miRNAs	Representative miRNAs	*p*-Value/FDR
Heart development	251	7	hsa-miR-17-5p, hsa-miR-22-3p, hsa-miR-24-3p, hsa-miR-27a-3p, hsa-miR-29a-3p, hsa-miR-125b-5p, hsa-miR-106b-5p	*p* = 2.53 × 10^−30^; FDR = 2.40 × 10^−29^

**Table 3:** PCR conditions.

Step	Temperature	Time	Cycles
Enzyme activation	95 °C	20 sec	1
Denature	95 °C	3 sec	40
Anneal/Extend	60 °C	30 sec	40

**Table 4:** List of pre-designed probes-TaqMan Advanced miRNA assay.

**Gene**	**Origin**	**Assay ID**
hsa-miR-191-5p	Thermo Fisher Scientific	477952_mir
hsa-miR-17-5p	Thermo Fisher Scientific	478447_mir
hsa-miR-451a	Thermo Fisher Scientific	478107_mir
hsa- miR-29a-3p	Thermo Fisher Scientific	478587_mir

## Data Availability

The original contributions presented in this study are included in the article/Supplementary Materials. Further inquiries can be directed to the corresponding author.

## Supplementary Materials

The following supporting information can be downloaded at: https://www.mdpi.com/article/10.3390/ijms262210813/s1.

## Abbreviations

The following abbreviations are used in this manuscript:

BAV	Bicuspid Aortic Valve
CAVD	Calcific Aortic Valve Disease
DOACs	Direct Oral Anticoagulants
e-GRF	Estimated Glomerular Filtration Rate
GO	Gene Ontology
PCR	Polymerase Chain Reaction
miRNA	Micro RNA
qRT-PCR	Quantitative Real-Time Polymerase Chain Reaction
SAVR	Surgical Aortic Valve Replacement
VEGFA	Vascular Endothelial Growth Factor A
VSMC	Vascular Smooth Muscular Cell
