# Supplementary material for: Molecular Network Analysis of Circulating microRNAs Highlights miR-17-5p and miR-29a-3p as Potential Biomarkers of Aortic Valve Calcification
Source: Int J Mol Sci. 2025 Nov 7;26(22):10813. doi: 10.3390/ijms262210813 (PMC12652765; doi:10.3390/ijms262210813)
Supplement: Supplementary file 1 [file ijms-26-10813-s001.zip › ijms-3967691-supplementary.pdf]

SupplementaryTable S1

miRNA	miRBase_MIMAT	Sequence	Length	Notes
hsa-miR-17-5p	MIMAT0000070	CAAAGUGCUUACAGUGCAGGUAG	23	miRBase v22.1
hsa-miR-17-3p	MIMAT0000071	ACUGCAGUGAAGGCACUUGUAG	22	miRBase v22.1
hsa-miR-20a-5p	MIMAT0000075	UAAAGUGCUUAUAGUGCAGGUAG	23	miRBase v22.1
hsa-miR-22-3p	MIMAT0000077	AAGCUGCCAGUUGAAGAACUGU	22	miRBase v22.1
hsa-miR-24-3p	MIMAT0000080	UGGCUCAGUUCAGCAGGAACAG	22	miRBase v22.1
hsa-miR-25-3p	MIMAT0000081	CAUUGCACUUGUCUCGGUCUGA	22	miRBase v22.1
hsa-miR-26a-5p	MIMAT0000082	UUCAAGUAAUCCAGGAUAGGCU	22	miRBase v22.1
hsa-miR-26b-5p	MIMAT0000083	UUCAAGUAAUUCAGGAUAGGU	21	miRBase v22.1
hsa-miR-27a-3p	MIMAT0000084	UUCACAGUGGCUAAGUUCCGC	21	miRBase v22.1
hsa-miR-29a-3p	MIMAT0000086	UAGCACCAUCUGAAAUCGGUUA	22	miRBase v22.1
hsa-miR-92a-3p	MIMAT0000092	UAUUGCACUUGUCCCGGCCUGU	22	miRBase v22.1
hsa-miR-106b-5p	MIMAT0000680	UAAAGUGCUGACAGUGCAGAU	21	miRBase v22.1
hsa-miR-125b-5p	MIMAT0000423	UCCCUGAGACCCUAACUUGUGA	22	miRBase v22.1
hsa-miR-140-3p	MIMAT0004597	UACCACAGGGUAGAACCACGG	21	miRBase v22.1
hsa-miR-143-3p	MIMAT0000435	UGAGAUGAAGCACUGUAGCUC	21	miRBase v22.1
hsa-miR-145-5p	MIMAT0000437	GUCCAGUUUUCCCAGGAAUCCCU	23	miRBase v22.1
hsa-miR-148a-3p	MIMAT0000243	UCAGUGCACUACAGAACUUUGU	22	miRBase v22.1
hsa-miR-423-5p	MIMAT0004748	UGAGGGGCAGAGAGCGAGACUUU	23	miRBase v22.1
hsa-miR-451a	MIMAT0001631	AAACCGUUACCAUUACUGAGUU	22	miRBase v22.1

Supplementary Table S2

Target Gene IDs	Target Gene Names	Targets Resource
ENSG00000115977	AAK1	TarBase v8.0
ENSG00000075624	ACTB	TarBase v8.0
ENSG00000184009	ACTG1	TarBase v8.0
ENSG00000138071	ACTR2	TarBase v8.0
ENSG00000006125	AP2B1	TarBase v8.0
ENSG00000165527	ARF6	TarBase v8.0

ENSG00000001626	CFTR	TarBase v8.0
ENSG00000141367	CLTC	TarBase v8.0
ENSG00000085832	EPS15	TarBase v8.0
ENSG00000109971	HSPA8	TarBase v8.0
ENSG00000205726	ITSN1	TarBase v8.0
ENSG00000130164	LDLR	TarBase v8.0
ENSG00000138760	SCARB2	TarBase v8.0
ENSG00000147010	SH3KBP1	TarBase v8.0
ENSG00000159082	SYNJ1	TarBase v8.0
ENSG00000152291	TGOLN2	TarBase v8.0
ENSG00000117533	VAMP4	TarBase v8.0
